# Evaluation of genotype MTBDR*plus* VER 2.0 line probe assay for the detection of MDR-TB in smear positive and negative sputum samples

**DOI:** 10.1186/s12879-017-2389-6

**Published:** 2017-04-17

**Authors:** Abyot Meaza, Abebaw Kebede, Zelalem Yaregal, Zekarias Dagne, Shewki Moga, Bazezew Yenew, Getu Diriba, Helina Molalign, Mengistu Tadesse, Desalegn Adisse, Muluwork Getahun, Kassu Desta

**Affiliations:** 1 Ethiopian Public Health Institute, Addis Ababa, Ethiopia; 2 Addis Ababa University College of Health Science Department of Medical Laboratory Science, Addis Ababa, Ethiopia

**Keywords:** Performance, Genotype MTBDR*plus* VER 2.0, MDR-TB

## Abstract

**Background:**

Multi drug resistant tuberculosis (MDR-TB) poses formidable challenges to TB control due to its complex diagnostic and treatment challenges and often associated with a high rate of mortality. Accurate and rapid detection of MDR-TB is critical for timely initiation of treatment. Line Probe Assay (LPA) is a qualitative in vitro diagnostic test based on DNA-STRIP technology for the identification of the M. *tuberculosis* complex and its resistance to rifampicin (RMP) and/or isoniazid (INH). Hain Lifescience, GmbH, Germany has improved the sensitivity of Genotype MTBDR*plus* VER 2.0 LPA for the detection of MDR-TB; with the possibility of applying the tool in smear negative sputum samples.

**Method:**

A cross sectional study was conducted on 274 presumptive MDR-TB patients referred to the National TB Reference Laboratory (NTRL), Ethiopian Public Health Institute (EPHI) who submitted sputum samples for laboratory diagnosis of drug resistant-TB testing. Seventy-two smear and culture positive samples processed in smear positive direct LPA category and 197 smear negative sputum samples were processed for direct LPA. Among the smear negative samples 145 (73.6%) were culture negative and 26 (13.2%) were culture positive. All specimens were processed using NALC-NaOH method and ZN smear microscopy done from sediments. Genotype MTBDR*plus* VER 2.0 done from processed sputum sediments and the result was compared against the reference, BACTEC MGIT 960 culture and DST. Sensitivity, specificity, PPV and NPV of Genotype MTBDR*plus* VER 2.0 assay was determined and *P*-value <0.05 was considered as statistically significant.

**Results:**

The sensitivity, specificity, PPV and NPV of Genotype MTBDR*plus* VER 2.0 LPA were 96.4, 100, 100 and 96.9%, respectively for the detection of MDR-TB from direct smear positive sputum samples. The sensitivity, specificity, PPV and NPV of Genotype MTBDR plus VER 2.0 LPA were 77.8, 97.2, 82.4 and 97.2%, respectively, for the detection of *M. tuberculosis* from direct smear negative sputum samples. Fourteen (53.8%) samples had valid results with LPA among the 26 smear negative culture positive samples. The remaining 8 (30.8%) and 4 (15.4%) were invalid and negative with LPA, respectively. The sensitivity and specificity of Genotype MTBDR*plus* VER 2.0 LPA were 100% for the detection of MDR-TB among 14 direct smear negative and culture positive sputum samples.

The most common mutations associated with RMP and INH resistance were S531L and S315TL, respectively. A single rare mutation (C15T/A16G) was detected for INH resistance.

**Conclusion:**

The diagnostic performance of Genotype MTBDR*plus* VER 2.0 LPA in direct smear positive sputum sample was highly sensitive and specific for early detection of MDR-TB. However, the diagnostic performance of this molecular assay in direct smear negative sputum sample was low and showed a high level of invalid results for detection of *M. tuberculosis* and its resistance to RMP and/or INH so it is unlikely to implement Genotype MTBDR*plus* VER 2.0 for the detection of MDR-TB in direct smear negative sample in our routine settings. The sensitivity of the assay should be improved for detection of MDR-TB in direct smear negative sputum specimens.

## Background

Tuberculosis (TB) remains a major global health problem, responsible for ill health among millions of people each year. TB ranks as the second leading cause of death from an infectious disease worldwide, after the HIV. The latest estimates included in this report are that there were 9.0 million new TB cases in 2013 and 1.5 million TB deaths (1.1 million among HIV-negative people and 0.4 million among HIV-positive people). TB mortality is unacceptably high given that most deaths are preventable if people can access health care for a diagnosis and the correct treatment is provided [1, 2].

According to the 2011 Ministry of Health report, TB is the eighth leading cause of hospital admissions and the third leading cause of hospital deaths in Ethiopia. In 1992, to prevent and limit the spread of the disease in Ethiopia, the government implemented the DOTS Strategy, the backbone of global TB control, whose objectives are the diagnosis of 70% of new smear positive TB cases and achieving 85% cure [3].

The first population-based national tuberculosis prevalence survey in Ethiopia which was done in 2010–2011 showed that the prevalence of smear-positive TB and bacteriologically confirmed TB were 108/100000 (95%CI 73–143), and 277/100000 (95%CI 208–347) respectively. The finding indicated that the actual TB prevalence in Ethiopia was much lower than the World Health Organization (WHO) estimates [3].

Globally in 2013, data from drug resistance surveys and continuous surveillance among notified TB cases suggest that 3.5% of newly diagnosed TB cases and 20.5% of those previously treated for TB had MDR-TB. The highest levels of MDR-TB are found in Eastern Europe and central Asia, where in some countries more than 20% of new TB cases and more than 50% of those previously treated for TB have MDR-TB [1].

The first countrywide anti-tuberculosis drug resistance survey in Ethiopia was carried out between 2003 and 2006. The survey reported the levels of MDR-TB in new and in previously treated patients, as 1.6% and 11.8% respectively [4].

The second round national anti-tuberculosis drug resistance surveillance in Ethiopia also done from November 2011 to June 2013. Multidrug resistant isolates were detected in 80 of the total 1651 samples from new and previously treated cases, making the overall prevalence of MDR-TB was 4.8%. The prevalence of MDR-TB among survey participants was 2.3% and 17.8% among new and previously treated cases, respectively [5].

Conventional drug susceptibility testing using a solid medium such as Lowenstein – Jensen is time consuming, whereas liquid medium based methods such as Mycobacterium Growth Indicator Tube (MGIT) system are sensitive but expensive. New assay such as Line Probe Assay (LPA) have been developed to detect resistance faster and reliable using genotype rather than phenotype and have been endorsed by WHO for the fast and reliable detection M. *tuberculosis* complex and its resistance to RMP and/or INH [6].

The Genotype Mycobacterium tuberculosis drug resistant (MTBDR) *plus* VER 2.0 is a qualitative in vitro test for the identification of the M. *tuberculosis* complex and its resistance to rifampicin and/or isoniazid from pulmonary smear positive and negative clinical specimens and cultivated isolates. This molecular genetic assay is based on DNA-STRIP technology and it includes DNA extraction, master mix preparation and addition, multiplex amplification with biotinylated primers and detection with reverse hybridization. The test is an aid in the rapid diagnosis of MDR-TB which is a prerequisite for the appropriate treatment initiation [6].

The aim of this study was to evaluate and provide information about the diagnostic performance of Genotype MTBDR*plus* VER 2.0 LPA for the detection of drug resistance TB in smear positive and negative sputum samples comparing against conventional liquid culture based reference standard method, BACTEC MGIT 960 culture and DST. This study also provides information on effective use of the test and/or for further recommendations in smear negative sputum samples in routine laboratory service. Evaluating new molecular tools such as Genotype MTBDR*plus* VER 2.0 LPA offer opportunity to scale up DST capacity in Ethiopia.

## Methods

A cross sectional study was conducted on presumptive MDR-TB patients referred to the NTRL, Ethiopian Public Health Institute(EPHI) from April to August 2015 in our routine laboratory setting. The Genotype MTBDR*plus* VER 2.0 assay was compared against conventional liquid culture based reference standard method, BACTEC MGIT 960 culture and DST.

### Sample size

Sputum samples were collected from Presumptive MDR-TB patients who were >15 years of age with consent based on non-probability convenience sampling technique [7] and calculated as followed below.

N = (zα/2)^2^p(1 − P)/d^2^

N = Number of individuals to be participated in the research,

z = standard normal distribution curve value for 95% confidence level, zα/2 = 1.96.

d = margin of error taken as 5%.

P = sensitivity of the assay, 97%(0.97).

*N* = 194, Sample size.

In standard culturing technique there is culture contamination and culture negative with acceptable range, However MGIT culture is more prone to culture contamination and there was low record that smear negatives turned to be culture positive so for each, 20% contingency added and this would compensate the samples that lost by contamination and culture negativity.

$$ \begin{array}{l}\mathrm{Contingency}\ \mathrm{or}\ \mathrm{culture}\ \mathrm{contamination},20\%=40\ \mathrm{and}\ \mathrm{Contingency}\ \mathrm{for}\ \mathrm{culture}\ \mathrm{negatives},20\%=40\hfill \\ {}\mathrm{Total}= N+\mathrm{total}\ \mathrm{contingency}=194+80\hfill \\ {}\mathrm{Total}=274\hfill \end{array} $$

A total of 274 samples collected for the study considering contingency for samples that would be lost by culture contamination and culture negativity.

After AFB smear microscopy done from processed sediment, the sample would be categorized in to smear negative and smear positive sputum sample to process LPA accordingly. After smear microscopy we found 72 smear positive and 202 smear negative sputum sample which would be processed for direct smear positive LPA and smear negative LPA, respectively in the study.

### Specimen collection, storage and transportation

Prior to collection of specimen, eligible study participants were signed on the consent form and basic demographic data and clinical information concerning the previous history of TB checked and obtained from the request form. Based on the criteria for Presumptive MDR-TB and Programmatic Management of Drug resistant Tuberculosis (PMDT) [8] who satisfied the study inclusion criteria were asked to provide one morning 5 ml sputum sample in a sterile screw cap universal disposable container provided for the routine diagnosis. The sputum samples were kept in a refrigerator at a maximum temperature of +4^0^c until the specimens were processed for culture within two days of delivery.

### Laboratory investigation

#### Sample processing, inoculation and smear microscopy

**Fig. 1:**
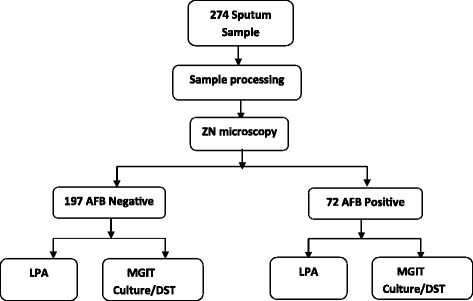
Flow diagram for AFB negative and AFB positive sample for laboratory investigation of LPA and MGIT culture and DST. *ZN-Zeihl Nelson, MGIT-Mycobacterium Growth Indicator Tube, LPA-Line Probe AssayNote: 5 samples lost during sample processing

We processed 274 sputum samples for digestion, decontamination and concentration (Fig. 1). Equal volumes of sputum and reagents (NaoH-Nacitrate with NALC) added to 50 ml falcon tube up to 10 ml inside biological safety cabinet (BSC) and mixed with vortex for 20 sec incubated for 15 min. After addition of 35 ml of Phosphate buffer solution the mixture centrifuged for 15 min at 3000 g in 4^0^c safety centrifuge. Smear preparation was made from the sediment with a sterile loop for AFB microscopy. Then the sediment was neutralized and resuspended in a 1 ml phosphate buffer solution and inoculated to Liquid media (Middle brook 7H9 broth base) in which PANTA and Supplement were added for the growth of the bacilli. MGIT 96o machine loaded with the inoculated media for the incubation and growth of the culture. For quality control, start and control sterile distilled water were processed as a sample to ensure the quality of sample processing using start and end control. Smears which is prepared from the sediment were stained with Zeihl Nelson and stained smears were read with light microscopy [9–11].

#### Identification

To differentiate whether the growth is due to contamination with other microorganisms or true *M. tuberculosis* complex growth, we performed blood agar inoculation and ZN smear for MGIT positive growths. The Rapid TB anigen (SD Bioline) was tested to confirm the presence of organisms belonging to M. tuberculosis complex once growth is observed in MGIT. Known positive and negative control strains were tested for Rapid TB antigen per new batch [9–11].

#### Line probe assay

DNA was extracted from the appropriate sample using chemical A and B inside BSC. After final centrifugation the supernatant was taken as DNA extract. Master mix was prepared in a clean room to prevent contamination of molecular laboratory. In addition room, 5 μl of DNA extracts was added to the corresponding PCR tubes, 5 μl of DNA extract from H37Rv quality control strain to the positive control tubes and 5 μl of distilled water the negative control tube. After addition, the mixture with the polymerase chain reaction (PCR) tube placed in to PCR machine for amplification. After completion of PCR process, the amplicon was detected with a series of procedures by adding different reagents to the strip. The strips were formed color bands after addition of the final substrate reagent [6].

#### MGIT DST

We performed MGIT DST by inoculating two MGIT tubes with the test culture. A known concentration of a test drug was added to one of the MGIT tubes, and growth was compared with the MGIT tube without the drug (growth control). Growth was monitored by the BACTEC 960 instrument which automatically interprets results as susceptible or resistant. One H37RV sensitivity strain was run per batch of DST set for quality control purpose [9–11].

### Data processing and statistical analysis

Statistical analysis was carried out using SPSS version 20 software packages. Sensitivity, specificity, PPV and NPV of the MTBDR*plus* VER 2.0 line probe assay was calculated among smear positive and negative sputum samples, comparing the results with the reference standard method, BACTEC MGIT 960 culture and DST and the results was interpreted based on 95% confidence interval, statistical significant was taken at *p*-value <0.05.

## Results

### Sociodemographic characteristics

A total of 274 subjects were included in this study. Among these 169 (61.7%) were male and the average age was 37 years. One hundred-thirty four (48.9%) had no previous history of TB treatment, 78 (28.5%) were relapse, 32 (11.7%) were return after default, 18 (6.5%) were treatment failure, 1 (0.4%) MDR-TB contact and 11 (4%) had unknown treatment history.

### Performance of LPA from smear positive sample

Seventy-two samples were smear positive and culture positive. Results of LPA were compared with results of Mycobacterium Growth Indicator Tube (MGIT) first line DST (Fig. 1). The sensitivity, specificity, PPV and NPV of Genotype MTBDR*plus* VER 2.0 LPA were 88.2 (30/34) 89.5 (34/38), 88.2 (30/34) and 89.5 (34/38) %, respectively for the detection of RMP resistance directly from smear positive sputum sample. The sensitivity, specificity, PPV and NPV of Genotype MTBDR*plus* VER 2.0 LPA were 91.7 (33/36), 97.2 (35/36), 97.1 (33/34) and 92.1(35/38) %, respectively for the detection of INH resistance directly from smear positive sputum sample. The sensitivity, specificity, PPV and NPV of Genotype MTBDR plus VER 2.0 LPA were 96.4(27/28), 100 (31/31), 100 (27/27) and 96.9 (31/32) %, respectively for the detection of MDR-TB directly from smear positive sputum sample (Table 1).

**Table 1:** Performance characteristics of Genotype MTBDR*plus* VER 2.0 LPA for detection RMP and INH resistance and MDR-TB in smear positive direct sample, 2015

Detection of drug resistance	MGIT R	MGIT S	LPA R	LPA S	Sensitivity%	Specificity%	PPV%	NPV%
					(95%* CI)	(95%* CI)	(95%* CI)	(95%* CI)
*RMP	34	38	34	38	88.2 (72.6–96.7)	89.5 (75.2–97.1)	88.2 (72.6–96.7)	89.5 (75.2–97.1)
*INH	36	36	34	38	91.7 (77.5–98.3)	97.2 (85.5–99.9)	97.1 (84.7–99.9)	92.1 (78.6–98.3)
*MDR-TB (RMP&INH)	28	31	27	32	96.4 (81.7–99.9)	100 (88.8–100)	100 (87.2–100)	96.9 (83.8–99.9)

*RMP Rifampicin, *INH* Isoniazid, *MDR*-*TB* Multidrug resistant Tuberculosis, *R* Resistant, *S* Susceptible, *CI* Confidence Interval

### Performance of LPA from smear negative direct sputum sample

**Fig. 2:**
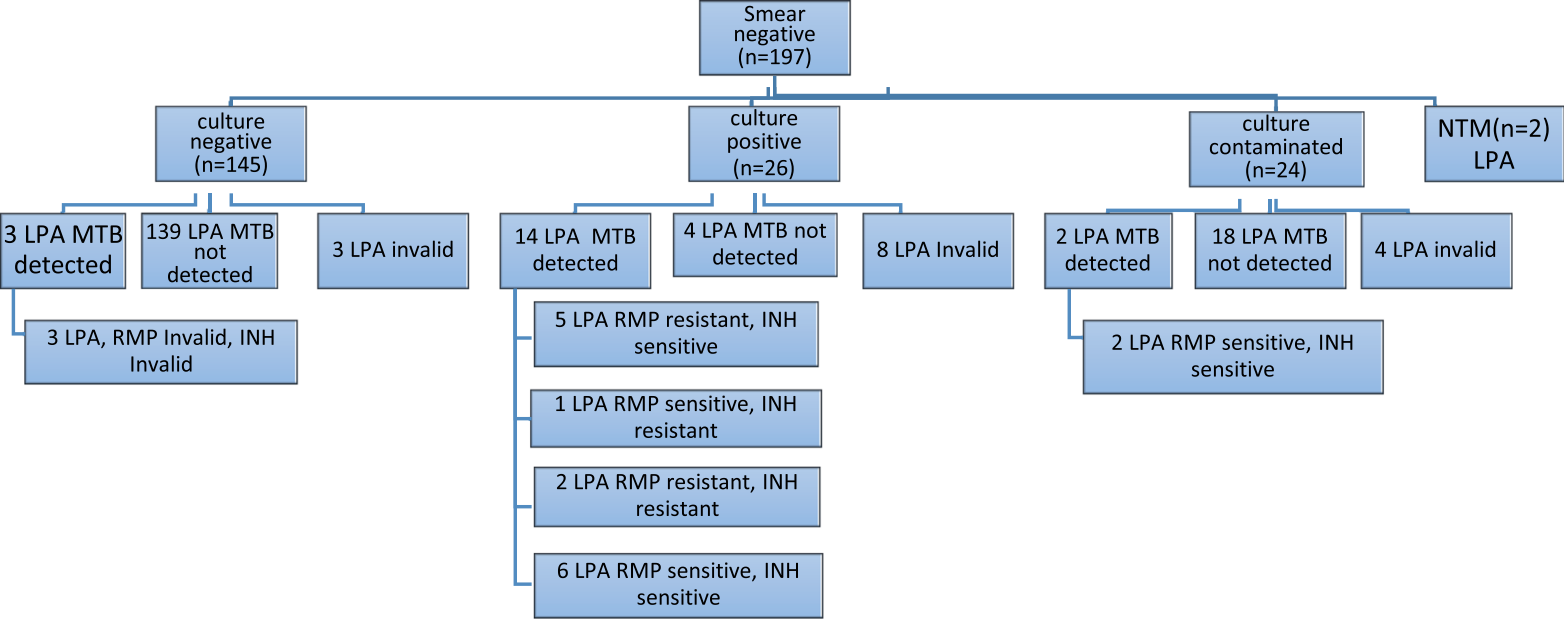
Performance of LPA from a total of 197 smear negative direct sputum sample. *LPA-Line Probe Assay, MTB - Mycobacterium Tuberculosis, NTM-Nontuberculous Mycobacterium

A total of 197 smear negative sputum samples were processed for direct LPA (Fig. 1) and from these 145(73.6%) were culture negative and 26(13.2%) were culture positive. Among 145 smear negative and culture negative samples, LPA results were negative in 139 (96%), Invalid in 3 (2%) and falsely detected in 3 (2%) of the sample (Fig. 2).

The sensitivity of Genotype MTBDR*plus* VER 2.0 LPA found to be 77.8 (14/18) % for the detection of *M. tuberculosis* from direct smear negative sputum sample (Table 2).

**Table 2:** Performance characteristics of Genotype MTBDR *plus* VER 2.0 LPA for detection *M. tuberculosis* in smear negative sample, 2015

MGIT positive	MGIT negative	LPA positive	LPA negative	Sensitivity%	Specificity%	PPV%	NPV%
				(95%* CI)	(95%* CI)	(95%* CI)	(95%* CI)
18	142	17	143	77.8 (52.4–93.6)	97.9 (94–99.6)	82.4 (56.6–96.2)	97.2 (93–99.2)

*CI Confidence Interval

Among the 26 smear negative and culture positive sample the LPA had valid results in 14 of the samples for the detection of RMP and INH resistance. Low specificity and PPV was found for the detection of RMP resistance directly from smear negative and culture positive sputum sample. The sensitivity of Genotype MTBDR plus VER 2.0 LPA was low, 60 (3/5) % for the detection of INH resistance directly from smear negative and culture positive sputum sample. The sensitivity, specificity, PPV and NPV of Genotype MTBDR*plus* VER 2.0 LPA were 100 (2/2), 100 (5/5), 100 (2/2) and 100 (5/5) % respectively, for the detection of MDR-TB directly from smear negative and culture positive sputum sample (Table 3).

**Table 3:** Performance characteristics of Genotype MTBDR*plus* VER 2.0 LPA for detection RMP and INH resistance and MDR-TB in smear negative and culture positive direct sample, 2015

Detection of drug resistance	MGIT R	MGIT S	LPA R	LPA S	Sensitivity%	Specificity%	PPV%	NPV%
					(95%* CI)	(95%* CI)	(95%* CI)	(95%* CI)
*RMP	3	11	7	7	100 (29.2–100)	63.6 (30.8–89.1)	42.9 (9.9–81.6)	100 (59–100)
*INH	5	9	3	11	60 (14.7–94.7)	100 (66.4–100)	100 (29.2–100)	81.8 (48.2–97.7)
*MDR-TB (RMP&INH)	2	5	2	5	100 (15.8–100)	100 (47.8–100)	100 (15.8–100)	100 (47.8–100)

*RMP Rifampicin, *INH* Isoniazid, *MDR*-*TB* Multidrug resistant Tuberculosis, *CI* Confidence Interval

### Mutations associated with RMP and INH drug resistant TB

The frequency of mutations associated with RMP and INH drug resistant TB analysed among 35 results of Genotype MTBDR*plus* VER 2.0 and concordant with MGIT DST.

Twenty seven (77.1%) were missing of wild type 8 (530–533) and mutation S531L in *rpoB* gene and this was the most frequent mutation associated with RMP resistance. On the other hand 28 (80%) were missing wild type (315) and mutation S315TL in *KatG* gene and this was the most frequent mutation associated with INH resistance. A single rare mutation (C15T/A16G) in *inhA* gene was detected in this study (Table 4).

**Table 4:** Mutations associated with RMP and INH drug resistant TB among concordant Resistant result with Genotype MTBDR*plus* VER 2.0 and MGIT DST

RMP resistance		INH resistance				
*rpoB* gene		*KatG* gene		*inhA* gene		
WT1–8 missing	Mutant	WT missing	Mutant	WT1–2 missing	Mutant	Frequency
530–533 (WT8)	S531L	315	S315TL	-	-	23
530–533 (WT8)	S531L	315	-	-	-	3
526–529 (WT7)	H526Y	315	S315TL	-	-	1
530–533 (WT8)	S531L	315	-	-	-	1
526–529 (WT7)	H526Y	315	S315TL	-15,-16 (WT1)	C15T	1
				-8 (WT2)	A16G	
^a^510–513 (WT2)	-					1
516–519 (WT4)						
522–526 (WT6)						
526–529 (WT7)						
^a^505–509 (WT1)	-					1
513–517 (WT3)						
526–529 (WT7)						
530–533 (WT8)						
		^b^315	S315TL	-	-	3
		^b^315	-	-15,-16 (WT1)	-	1

^a^Mutations associated with RMP mono resistance, ^b^Mutations associated with INH mono resistance

## Discussion

In this study the diagnostic performance of Genotype MTBDR*plus* VER 2.0 LPA was high for the detection of MDR-TB (sensitivity-96.4, specificity-100%) in smear positive sputum sample. Similar studies have reported high sensitivity and specificity for the detection of MDR-TB in smear positive sputum sample in Uganda by Albert et al. [12], India by Maduri et al. [13], Thailand by Anek et al. [14] and Moldova by Crudu et al. [15]. Only one sample (1.4%) detected as falsely susceptible for detection of MDR-TB and the same result was also detected from Xpert MTB/RIF assay. As both tests share a similar principle of molecular technique and there might be mutations present out of *rpoB* gene region, one falsely RMP sensitive result has been detected and this could be explained by the fact that, about 5% and 10% to 25% of resistant strains are thought to have mutations outside *rpoB* and, *katG* - *inhA* loci respectively [16].

In this study relatively high sensitivity and specificity was observed for the detection of RMP (sensitivity-88.2 and specificity-89.5%) and INH (sensitivity-91.7 and specificity-97.2%) resistance. Higher sensitivity and specificity reported for the detection of both RMP and INH resistance in a study done in India by Raizada et al. [17] and Germany by Hillemann et al. [18]. The reason for higher sensitivity and specificity could be due to large sample size used in the study by Raizada et al. [17] and gene sequencing used as reference standard to characterize the genotype of resistance mutations in the study by Hillemann et al. [18]. A systematic review and meta-analysis of 14 comparisons reviewed by Ling et al. [19] were also identified very high and consistent pooled sensitivity and specificity for the detection of RMP and INH resistance in smear positive sample. On the other hand lower sensitivity reported in Brazil by Maschmann et al. [16] and in India by Singhal et al. [20] for INH. This could be explained by the fact that, it is well known that about 10% to 25% of INH resistant strains are thought to have mutations outside *katG* and *inhA* loci [16].

The performance of LPA from smear negative sample showed low detection and similar report observed in the USA by Luetkemeyer et al. [21] that evaluated LPA for the detection of MTB and resistance to RMP and INH found very low sensitivity (44.1%) for AFB smear negative specimen however relatively high sensitivity (79.8%) reported in Brazil by Maschmann et al. [16]. This might be due to appropriate selection of the study population that used sputum of patients who had treatment failure or relapse in a routine outpatient setting in the study. In addition, in the study done in South Africa by Barnard et al. [22] to evaluate LPA with Xpert MTB/RIF from smear positive and negative sample reported that the diagnostic performance of the GenoType MTBDR*plus* VER 2 LPA was equivalent to that of the Xpert MTB/RIF assay. This could be retreatment cases were selected given their high risk of associated drug resistance and both assays were molecular techniques and share the same principle of testing that detect mutations conferring in *rpoB* gene region for the detection of RMP resistance.

Among the 26 smear negative sample and had culture positive growth, LPA detected valid result in 14(53.8%), negative in 4(15.4) and invalid in 8(30.8%) samples and this suggests that detection of *M. tuberculosis* and its resistance to RMP and/or INH prone high level of invalid results. In this study, high sensitivity and specificity observed in smear negative sample and which had confirmed culture growth for the detection of MDR-TB, however there were few number of valid results (2 for sensitivity and 5 for specificity) for comparison from other studies. In addition, high level of invalid and false negative results observed.

The most common mutation associated with RMP by Raizada et al. [17] was similar to the present study. This finding was also common with findings in a study done in Uganda by Albert et al. [12].

## Conclusion

The diagnostic performance of Genotype MTBDR*plus* VER 2.0 LPA in direct smear positive sputum sample was highly sensitive and specific for early detection of MDR-TB. The diagnostic performance of Genotype MTBDR*plus* VER 2.0 LPA in direct smear negative sputum sample was low and showed a high level of invalid results for detection of *M. tuberculosis* and its resistance to RMP and/or INH so it is unlikely to implement Genotype MTBDR*plus* VER 2.0 for the detection of MDR-TB in direct smear negative sample in our routine settings. The sensitivity of the assay should be improved for detection of MDR-TB in direct smear negative sputum specimens.

## Abbreviations

DNA: Deoxyribonucleic acid
DST: Drug Susceptibility Testing
EPHI: Ethiopian Public Health Institute
HIV: Human Immunodeficiency Virus
INH: Isoniazid
LPA: Line Probe Assay
M*. tuberculosis*: *Mycobacterium tuberculosis*
MDR TB: Multi drug resistance Tuberculosis
MGIT: Mycobacterium Growth Indicator Tube
MTBDR: *Mycobacterium tuberculosis* drug resistant
NPV: Negative Predictive Value
NTRL: National Tuberculosis Reference Laboratory
PMDT: Programmatic Management of Drug resistance Tuberculosis
PPV: Positive Predictive Value
RMP: Rifampicin
TB: Tuberculosis
WHO: World Health Organization
